# Dynamic impact response of lithium-ion batteries, constitutive properties and failure model†

**DOI:** 10.1039/c8ra08898e

**Published:** 2019-01-15

**Authors:** Golriz Kermani, Elham Sahraei

**Affiliations:** Electric Vehicle Safety Lab (EVSL), Department of Mechanical Engineering, Temple University Philadelphia PA 19122 USA elham.sahraei@temple.edu +1-215-204-4276; Department of Mechanical Engineering, Massachusetts Institute of Technology Cambridge MA 02139 USA; George Mason University, Fairfax VA 22030 USA

## Abstract

Use of lithium-ion batteries in transportation necessitates understanding of the cell mechanical response in case of a vehicle accident. Many researchers have access to test equipment to characterize the behavior of cells at low speeds. However, testing batteries at high speeds requires special setups that are not available for many interested parties. In this research, a methodology is proposed for predicting the material response and failure patterns of lithium-ion batteries subjected to high impact based on the experimental results at lower velocities. For this purpose, a constitutive law was proposed and parameters were calibrated for two types of lithium-ion cells, pouch and elliptical. Test results at lower impact velocities (up to 0.5 m s^−1^) were used for calibrating the constitutive response and the failure criteria. The test data at higher impact velocities of up to 5 m s^−1^ were used for validation. A Johnson–Cook type strain rate sensitivity model could successfully capture the strain rate hardening response of both cell types. Finite element models were developed for each cell type and empirical linear relationships were found between failure strain and strain rate. For the case of pouch cells, this correlation was negative, whilst there was a positive correlation for elliptical cells. The FE models closely followed the experiments in terms of load-displacement behavior and predicted the peak load and punch displacement at the onset of short circuit with good accuracy.

## Abstract 2

Dynamic response and failure of pouch and elliptical cells under impact loading.

## Graphical abstract

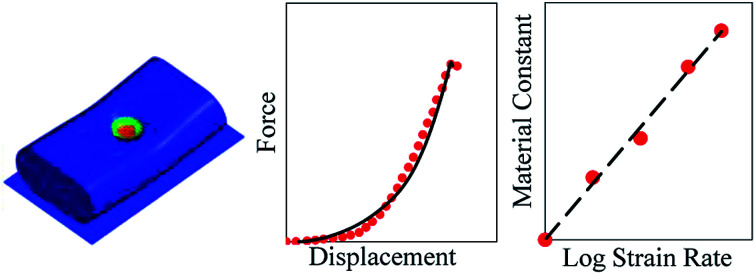

## 1. Introduction

During the past decade, lithium-ion batteries have been extensively used in electric vehicles, where they are subject to various loading scenarios ranging from vibrations under normal operation to crash pulses and impact loads during accidents. While the quasi-static response of such batteries under mechanical deformation has been studied in several recent publications, their behavior under dynamic loading is still not completely understood. It is of paramount importance to the automotive industry to characterize the cell response under high-speed loadings to gain better understanding of the underlying criteria/mechanisms of cell failure. Previous studies by Sahraei and co-workers have focused on characterization of large deformation and failure in pouch, cylindrical, and elliptical lithium-ion battery cells and components.^1–4^ Greve and Fehrenbach studied material and failure responses of large cylindrical cells under various loading scenarios.^5^ One of the main findings of these studies was that the mechanical failure coincides with the onset of short circuit and a drop in force. Pan and co-workers did extensive studies on testing and modeling of in-plane compression of lithium-ion pouch cells and modules.^6,7^ All of the above-mentioned studies focused on quasi-static behavior of the batteries.

Xu *et al.* developed a finite element (FE) model in ABAQUS based on the Greve and Fehrenbach experimental results on cylindrical cells. They assumed a strain rate dependent response of the jellyroll based on available rate dependent properties of aluminum and copper foils and extended their model to the dynamic regime. The FE models of cells were used for parametric studies; however, they were not validated against experimental data.^8^ In a later study, Xu *et al.* conducted drop tower tests with impact velocities of 2 to 3.5 m s^−1^ and calibrated/validated a new material model for jellyroll of cylindrical cells.^9^

Amodeo *et al.* have reported on dynamic testing of Representative Volume Element (RVE) specimens for pouch modules under in-plane compressive loads. A corresponding FE model was developed without accounting for the strain rate dependency. Higher stress levels were observed under dynamic loading as compared to quasi-static experiments and the FE models predicted the stress–strain response as well as the deformation.^10^

Kisters *et al.* have conducted extensive testing on lithium-ion pouch and elliptical cells.^11^ Their study reports on counter intuitive results showing a drop in failure resistance of pouch cells at higher speeds while opposite trends were observed for elliptical cells. To the best of the authors' knowledge, there is no available model to explain dynamic response of pouch and elliptical cells under local indentation, and no validated failure criterion exists to predict the onset of short circuit in lithium-ion cells under dynamic loads. Principle of virtual work has been previously used to characterize the mechanical properties of pouch cells under flat indentation.^3^ Quasi static flat compression tests on batteries can easily generate loads in the order of 200 kN. Equipment for dynamic testing usually do not have such high load capacities. Therefore, at high speeds, it is more feasible to perform hemispherical punch tests with small punch sizes where the maximum load remains under 20 kN. The first challenge of such tests is development of a methodology to calibrate mechanical properties of the cell from hemispherical punch tests rather than flat compression.

The objective of this study was to characterize the mechanical properties of pouch and elliptical cells under dynamic loading and to propose a failure criterion to detect the onset of electric short circuit for these batteries valid at high speeds. Selection of these two types of cells provide an understanding on whether a hard shell casing *versus* a pouch cover affects dynamic response of the cells. In the first step, an analytical model of the hemispherical punch indentation tests was suggested using the principle of virtual work. With this model, the material hardening curve was directly extracted from the corresponding hemispherical punch test data. The experimental results at lower strain rates were used to characterize the strain rate dependent constitutive material response of each battery cell type. Subsequently, finite element models were developed and used to validate the material properties. For the models, a failure criterion of maximum principal strain was considered. The failure strains were calibrated using hemispherical punch tests by matching the peak load and subsequent drop in the first three experiments (speeds lower than 0.5 m s^−1^). The evolution of failure strains as a linear function of strain rate was established. In the last stage, the experimental results at higher strain rates were used to validate both the constitutive model and the failure criterion.

## 2. Experiments

**Fig. 1:**
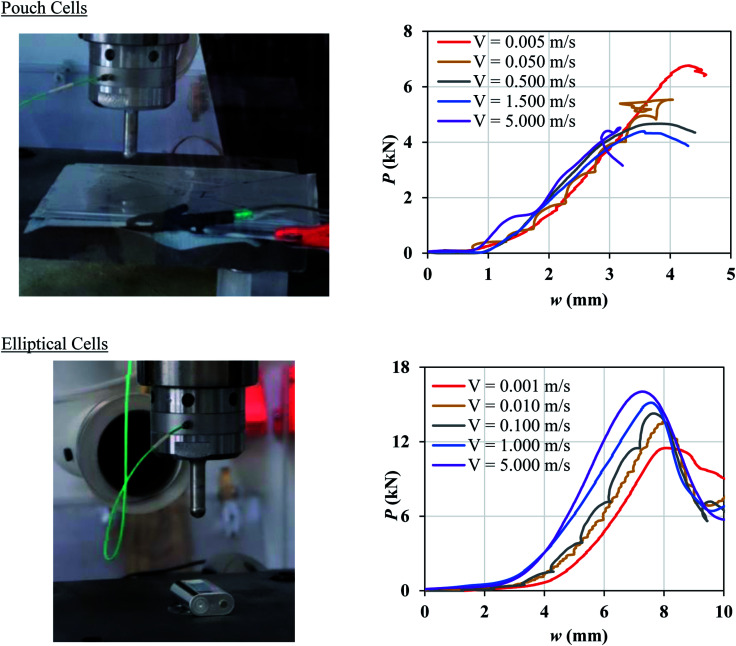
Experimental setup and force-displacement data of hemispherical punch indentation tests performed at EMI Fraunhofer Institute on a pouch cell (top) and an elliptical cell (bottom) as reported in Kisters *et al.*^11^

A number of dynamic impact tests were performed by Fraunhofer Ernst-Mach-Institute (EMI) in collaboration with the current authors. Details of the experimental setup, tested cells, and test results were reported by Kisters *et al.*^11^ Briefly, two types of pouch cells and one type of elliptical cell were impacted by a hemispherical punch of diameter 12.7 mm at various crosshead velocities up to 5 m s^−1^. Load, displacement, and cell voltage were recorded over time during all tests. Cell failure was identified by a drop in the voltage and the peak force. Flat compression tests could not be performed under dynamic loading, as the loads generated in such tests would go beyond the capacity of the load frame. In the current research, the test results at lower crosshead velocities in the range of 0.001 to 0.5 m s^−1^ were used to characterize the rate dependent material behavior and failure properties of the pouch and elliptical cells and the remaining tests (*V* ≥ 1 m s^−1^) were used to validate the models. Fig. 1 shows the two types of cells used in the experiments and the corresponding force-displacement (*P*–*w*) measurements for different impact velocities. The pouch cells were 329 mm × 161 mm × 12 mm and the elliptical cells were 37 mm × 64 mm × 18 mm. Readers are referred to by Kisters *et al.*^11^ for more details regarding the test setup and results.

## 3. Material characterization and calibration procedure

In previous research studies of the authors, the compressive stress–strain (*σ*–*ε*) curves for different batteries were calculated from a flat punch indentation or a compression between two flat plates.^3^ A flat compression creates a uniform state of stress under the punch; hence, the calculation of *σ*–*ε* curves from *P*–*w* data is rather straightforward in such loading scenarios. Flat compression tests at quasi-static loading usually generates very large forces (up to 200 kN). Current dynamic load frames do not have such capacities. Therefore, flat compression tests could not be performed at high speeds, and for characterizing the cell response under dynamic loading, only hemispherical punch indentations were possible. Therefore, it was important to develop a method to extract *σ*–*ε* relationship directly from *P*–*w* measurements from punch tests. For this purpose, the principle of virtual work was applied. For a virtual displacement of *δw*, one can write:1
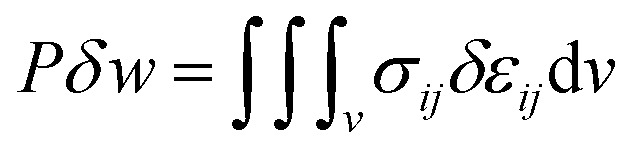


In the case of hemispherical punch tests, one can assume that the radial and circumferential components of strain are negligible due to low Poisson's ratio of battery components (active coatings and separators). As explained Wierzbicki and Sahraei 2013,^12^ the shear components are also negligible and the only major contribution comes from the through-thickness normal stress and strain. Therefore, the principle of virtual work reduces to:2
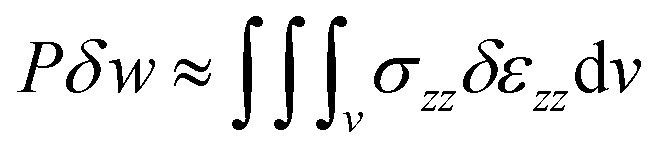


**Fig. 2:**
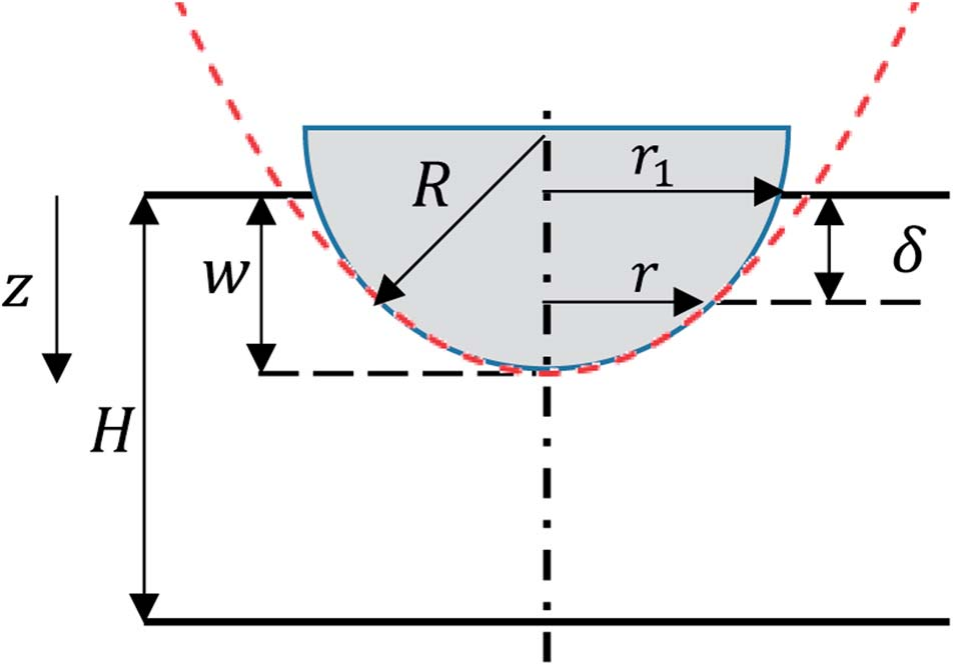
Schematic of deformation under a hemispherical punch. Red dashed line represents the parabolic function used to approximate the punch geometry.

Based on the previous studies of Sahraei *et al.*,^3^ the following power type constitutive model was assumed for the compressive stress–strain relationship:

3 *σ*_*zz*_ = *Aε*_*zz*_^*n*^

where *A* is the amplitude and *n* is the exponent. In the case of uniaxial strain, the strain is assumed to be constant in each column of the material under the punch in the *z* direction. This assumption was verified by detailed finite element modeling in our previous work.^12^ Therefore, the compressive strain can be calculated from:4
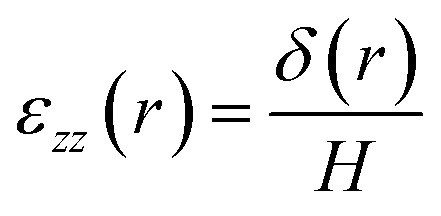
*H* is the cell thickness (*H* = 12 mm for pouch and *H* = 18 mm for elliptical cells) and *δ* is the vertical displacement of the material points under the punch, a function of distance from center of punch, *r*, as shown in Fig. 2.

Although the edge of deformation has a smooth transition to the hemispherical indented area, previous research has shown that the contribution from the transition area is minimal.^3^ Therefore, we can assume the shape of deformation as a parabola tangent to the hemispherical punch and *δ* can be written as:5
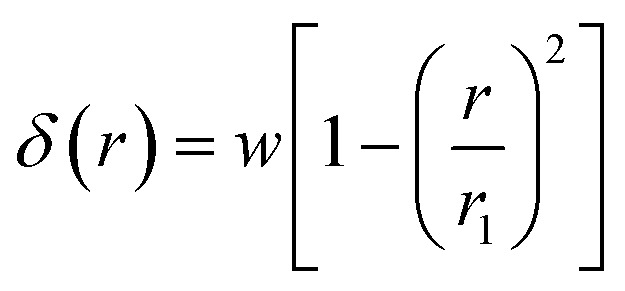
where *w* is the rigid punch displacement. *r*_1_ represents the radius of the contact area between the punch and the cell and can be expressed as 
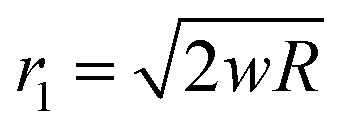
 for small indentation depths. *R* is the hemispherical punch radius and the volume element is d*v* = 2π(*H* − *δ*)*r*d*r*. Substituting eqn (3)–(5) into eqn (2) gives:6
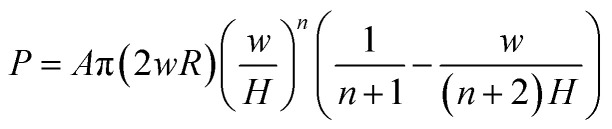


**Fig. 3:**
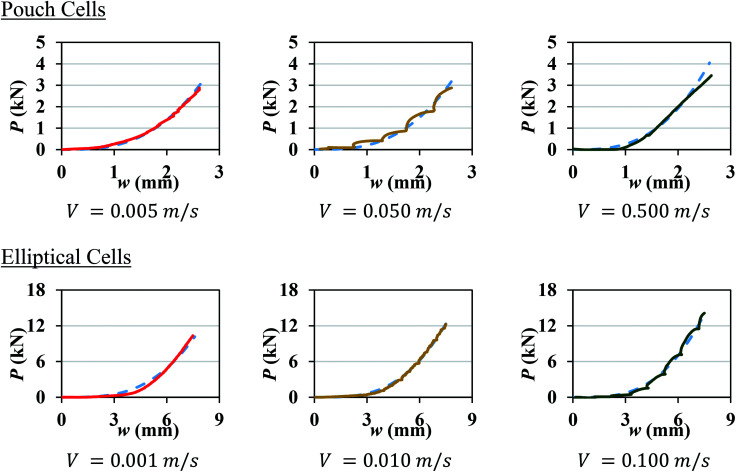
Analytical (dashed lines) *vs.* experimental (solid lines) load-displacement response of pouch cells (top) and elliptical cells (bottom) at different crosshead velocities

The experimental results up to impact velocities of 0.5 m s^−1^ for each cell were used to characterize the constitutive models. The experimental load-displacement curves were fitted by eqn (6) using MATLAB to calculate *A* and *n*, results of which are listed in Table 1 for pouch and elliptical cells. Analytical fits are compared to the experimental data in Fig. 3. It can be seen that the compressive strain–stress curves depend on the rate of indentation and can be written as *σ*(*ε*,*ε̇*) = *A*(*ε̇*)*ε*^*n*^ = *f*(*ε̇*)*σ*(*ε*).

**Table 1:** Constitutive model for pouch and elliptical cells

Pouch cells		Elliptical cells	
Crosshead velocity (m s^−1^)	*σ* = *A*(*ε̇*)*ε*^*n*^	Crosshead velocity (m s^−1^)	*σ* = *A*(**)*ε*^*n*^
0.005	*σ* = 2.17*ε*^2^	0.001	*σ* = 1.52*ε*^2.5^
0.050	*σ* = 2.34*ε*^2^	0.010	*σ* = 1.92*ε*^2.5^
0.500	*σ* = 3.00*ε*^2^	0.100	*σ* = 2.17*ε*^2.5^

Assuming a Johnson–Cook type model to describe the cells' rate dependent behavior, the above functions can be written as *f*(**) = (1 + *c* ln ***) and *σ*(*ε*) = (*A*_ref_*ε*^*n*^). Then:

7 *σ* = (*A*_ref_*ε*^*n*^)(1 + *c* ln ***)

This means:8
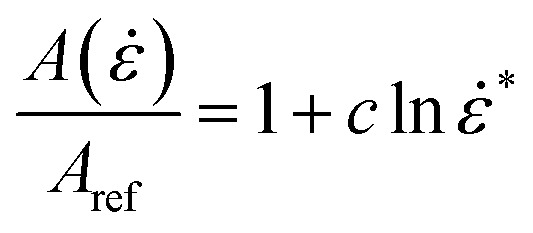
where *A*_ref_ is the value of the amplitude at quasi-static reference case and *c* is the strain-rate sensitivity parameter. *** = **/**_0_ is the normalized strain rate. **_0_ is the quasi-static strain rate and is equal to 0.4167 and 0.0556 s^−1^ for pouch and elliptical cells, respectively. The quasi-static tests had punch speeds of 0.005 and 0.001 m s^−1^ for pouch and elliptical cells, respectively. For pouch cells *A*_ref_ = 2.17 GPa and *c* = 0.0732 and for elliptical cells *A*_ref_ = 1.52 GPa and *c* = 0.0971.

## 4. Finite element simulation

LS Dyna software, a finite element program suitable for simulations of crash loading and large deformations, using explicit time integration, was used to simulate the dynamic impact tests on each cell. Interior of battery cells were modeled as homogenized parts using fully integrated solid elements.

The quasi-static stress–strain curves calculated from flat punch tests reported in^13,14^ were used in this study and inputted as piecewise curves in LS Dyna. For the strain rate effects, *f*(**) = (1 + *c* ln ***) was used to obtain the stress–strain curves at higher strain rates, while keeping the *σ*(*ε*) part of the curve as piecewise linear input. A modified crushable foam material model from library of LS Dyna, MAT_163, was used for both cells. MAT_ADD_EROSION was used to add failure in the model by a maximum principal strain (*ε*_*f*_) criterion. The hemispherical punch of diameter 12.7 mm was modeled with rigid shell elements. Tests at first three lower impact velocities were used to calibrate the values of failure strain for first three tests to capture the onset of short circuit, *i.e.*, the point at which there is a local peak in *P*–*w* curve.

### 4.1. Pouch cells

**Fig. 4:**
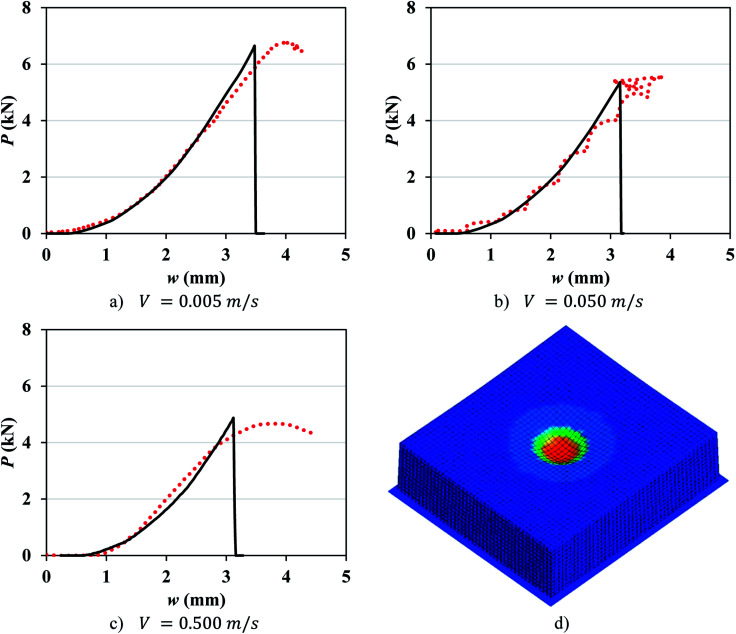
(a–c) Simulation with mesh 1.2 mm (solid lines) *vs.* experimental (dotted lines) load-displacement response of pouch cells at different crosshead velocities. (d) Typical stress distribution in the cell before failure.

The contribution of the cell casing was neglected in pouch cell models. The element size was 1.24 mm in the length and width directions and 1.06 mm in the thickness direction (mesh 1.2 mm). A mesh size of about 1 mm is chosen, as this is the most common mesh size used for full vehicle crash simulations. There were 11 594 elements in the pouch cell, only modeling the central part of the cell under the punch. The coefficient of friction was set to 0.3 between the cell and the punch, and 0.25 between the cell and the rigid wall. The number of failed integration points prior to element deletion (NUMFIP) was set to 2 and the number of cycles to determine the average volumetric strain rate (NCYCLE) was set to 18. Table 2 lists the values of *ε*_*f*_ for each crosshead speed. Comparison between simulation and test results for various crosshead velocities, as displayed in Fig. 4, shows good agreement in both the peak force and the indentation depth at the onset of failure.

**Table 2:** Calibrated failure strain, *ε*_*f*_, for pouch and elliptical cells

Pouch cell				Elliptical cell	
Crosshead velocity (m s^−1^)	*ε*_*f*_			Crosshead velocity (m s^−1^)	*ε*_*f*_
	Mesh 1.2 mm	Mesh 2.0 mm	Mesh 4.0 mm		Mesh 1.0 mm
0.005	0.075	0.109	0.118	0.001	0.060
0.050	0.068	0.100	0.105	0.010	0.130
0.500	0.059	0.093	0.100	0.100	0.105

**Fig. 5:**
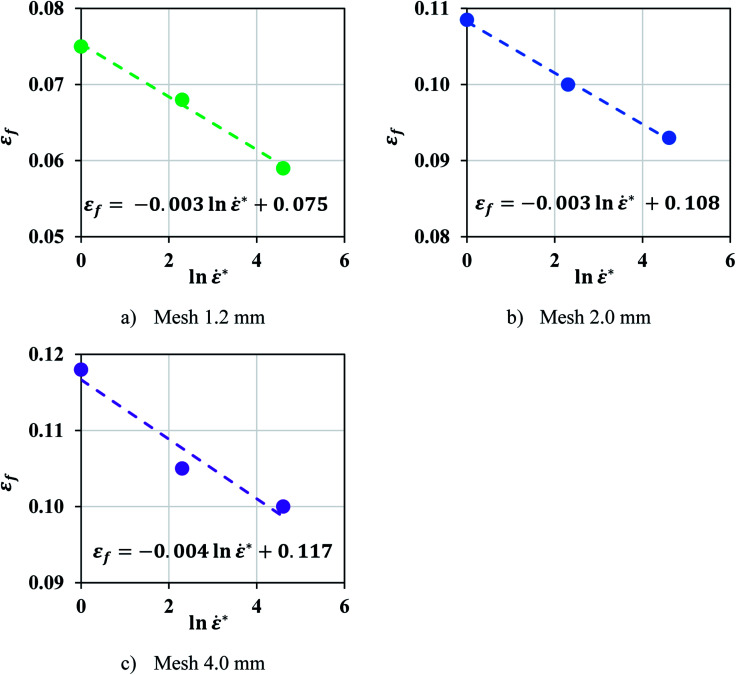
A negative linear relationship was found between *ε*_*f*_ and ln *** in pouch cell for all mesh sizes studied.

In order to study the effect of mesh size, coarser mesh sizes were developed: mesh 2.0 mm (1.92 × 2.11 × 1.95 mm) and mesh 4.0 mm (3.84 × 4.23 × 3.9 mm) and the values of *ε*_*f*_ were recalibrated (Table 2). It is observed that failure strain decreases as the strain rate increases with a linear correlation between *ε*_*f*_ and ln *** for pouch cells for all mesh sizes studied (Fig. 5). The following empirical linear relationships were found for each mesh size:

9a Mesh 1.2 mm: *ε*_*f*_ = −0.003 ln *** + 0.075

9b Mesh 2.0 mm: *ε*_*f*_ = −0.003 ln *** + 0.108

9c Mesh 4.0 mm: *ε*_*f*_ = −0.004 ln *** + 0.117

The use of coarse mesh sizes in the simulations significantly reduced the computational time and cost. This is specifically advantageous for modeling of industrial applications, such as electric vehicle battery modules and packs. Pouch cell model has proved to be stable even for large mesh sizes up to 4 mm which makes it a viable option for industrial use.

### 4.2. Elliptical cells

**Fig. 6:**
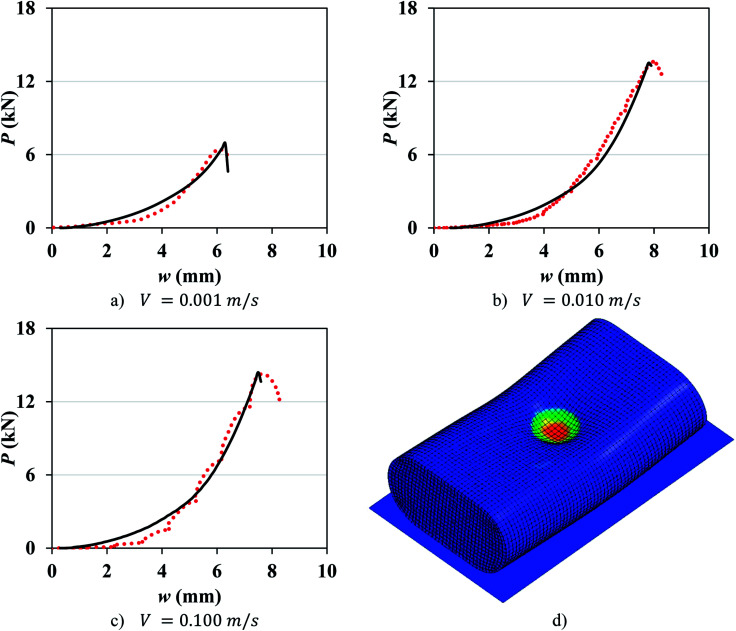
(a–c) Simulation (solid lines) *vs.* experimental (dotted lines) load-displacement response of elliptical cells at different crosshead velocities. (d) Typical stress distribution in the cell before failure.

The model for the elliptical cell consisted of three parts: jellyroll, shell casing, and endcaps. Jellyroll was modeled using 36 226 solid elements of 1 × 1 × 1 mm. The aluminum shell casing and two endcaps were modeled using shell elements with average element size of 1 mm and a piecewise linear plasticity material model (MAT_024). The rate dependency of aluminum shell casing was neglected in the current model. The interfaces between the cell components were modeled by eroding single surface contact and the coefficient of friction of 0.3. NUMFIP and NCYCLE were set to 2 and 18, respectively. Table 2 lists the values of *ε*_*f*_ and Fig. 6 shows a good agreement between simulations and dynamic tests at different rates for elliptical cells. Further analysis showed that there is a positive linear correlation between *ε*_*f*_ and ln ***:

10 *ε*_*f*_ = 0.010 ln *** + 0.076

### 4.3. Validation of the material and failure model at higher strain rates

**Fig. 7:**
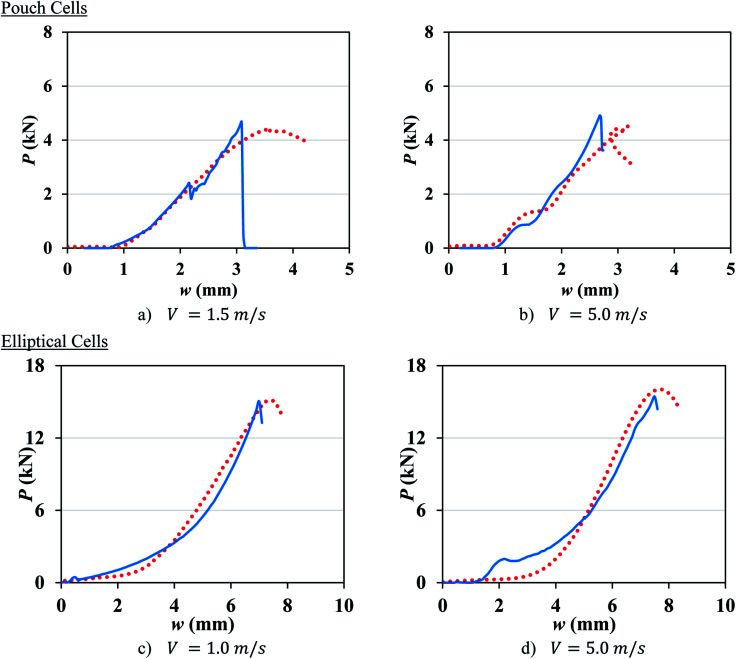
Model prediction (solid lines) *vs.* experimental (dotted lines) load-displacement response of pouch cells mesh 1.2 mm (top) and elliptical cells (bottom) at high crosshead velocities.

Experimental results at higher crosshead velocities for pouch (*V* = 1.5 and 5 m s^−1^) and elliptical (*V* = 1 and 5 m s^−1^) cells were used to validate the proposed constitutive model and the failure criteria. Eqn (8) was used to predict the constitutive model amplitude, *A*, at higher impact velocities for both cell types (Table 3). The values of *ε*_*f*_ for higher velocities were calculated from eqn (9) and (10), see Table 3. Fig. 7 compares the experimental results with the model predictions for pouch and elliptical cells. The models were successful in predicting the load-displacement curves and the onset of the failure (peak force) in the dynamic region with the maximum error of 10%.

**Table 3:** Model prediction of constitutive model parameter and failure strain for pouch and elliptical cells

Pouch cell					Elliptical cell		
Crosshead velocity (m s^−1^)	*A*(*ε̇*)	*ε*_*f*_			Crosshead velocity (m s^−1^)	*A*(*ε̇*)	*ε*_*f*_
		Mesh 1.2 mm	Mesh 2.0 mm	Mesh 4.0 mm			Mesh 1.0 mm
1.5	3.08	0.058	0.091	0.094	1.0	2.54	0.143
5.0	3.27	0.054	0.087	0.090	5.0	2.78	0.159

## 5. Discussion and conclusions

**Fig. 8:**
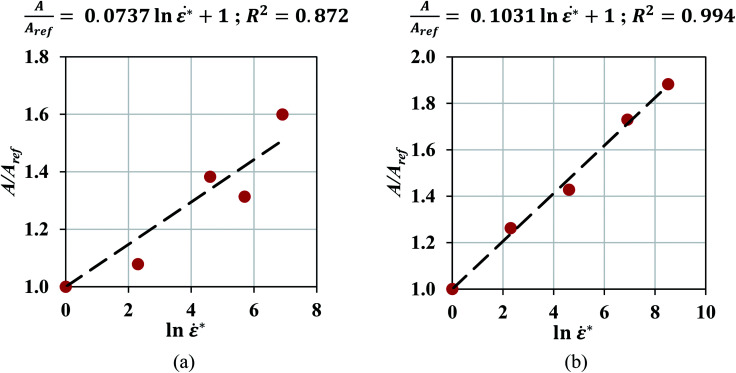
Linear relationship between the normalized fit coefficients and ln *** in (a) pouch and (b) elliptical cells.

**Fig. 9:**
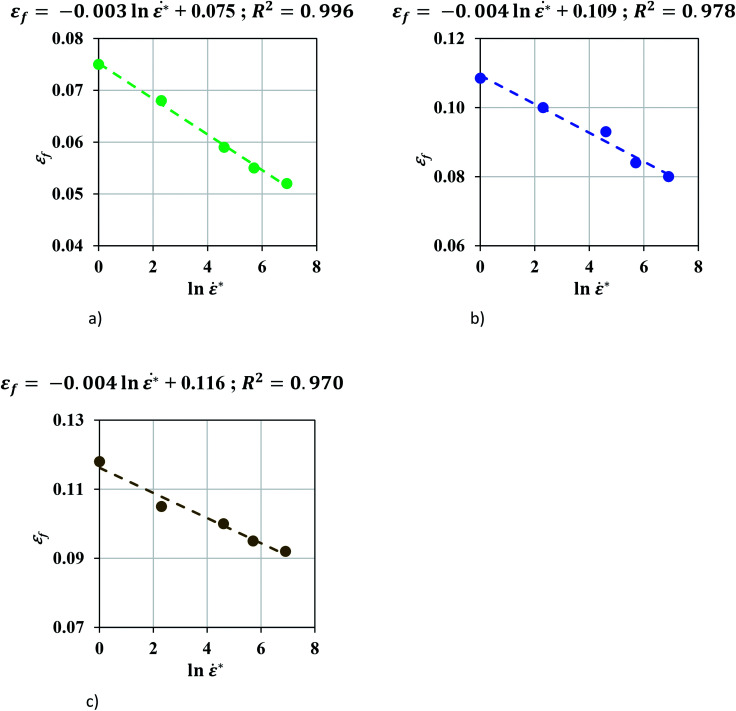
A negative linear relationship was found between *ε*_f_ and n *** in pouch cells for all mesh sizes studied (a–c). (a) Pouch cell, mesh 1.2 mm. (b) Pouch cell, mesh 2.0 mm. (c) Pouch Cell, mesh 4.0 mm.

The mechanical characteristics of pouch and elliptical cells subject to dynamic impact loads (up to impact velocities of 5 m s^−1^) and their corresponding failure criteria were investigated in this study. A method was proposed to extract material properties directly from hemispherical punch tests. Many dynamic load frames have limited capacity and cannot be used to test properties in uniform flat compression scenarios, where forces can go up to 200 kN. The proposed approach allows calibration of material model from hemispherical punch tests, where the maximum force is in the order of 10 kN. Results showed that a Johnson–Cook constitutive model of the form *σ* = (*A*_ref_*ε*^*n*^)(1 + *c* ln ***) successfully represented the hardening behavior of pouch and elliptical cells with *n* = 2 and 2.5, respectively. This trend was expected, since all cell components exhibit strain rate hardening behavior.^15–18^ Johnson–Cook model (*n* = 2.7) had been used previously in investigating the cylindrical cells under dynamic loadings.^8^ A finite element model was developed for each cell type to simulate the dynamic impact tests with a hemispherical punch and used to calibrate the maximum principal strain at various crosshead velocities. The models at lower speeds showed a linear trend for the failure strain as a function of strain rate. This data was used to estimate failure strain for higher speeds. Simulations of high-speed tests (at 1 to 5 m s^−1^) were used for validation of the models. The load-displacement response, the peak force, and the indentation depth at the onset of failure predicted by simulations were in good agreement with those from experiments. This suggests that the lithium-ion cell response in dynamic loading scenarios can be predicted based on the experiments conducted at a lower velocity range. To further verify the linear hardening behavior and the dependency of the failure criteria on strain rate, calibrations were repeated on high-speed test data without using the predictions of the models. Fig. 8 shows the linear relationship between the normalized fit coefficients (*A*/*A*_ref_) and ln *** for both pouch and elliptical cells and confirms this assumption is still valid when all tests are considered. Furthermore, the failure strains of pouch cells at high speeds were directly calibrated from experiments for all five tests using three mesh sizes and plotted in Fig. 9 which confirms the linear decreasing trend in failure strain over increasing strain rates.

Pouch and elliptical cells showed different mechanical response to dynamic loading. Pouch cells experienced lower force levels and smaller punch displacements at the onset of failure as compared to elliptical cells. In the case of pouch cells, *ε*_*f*_ decreased linearly with ln ***. On the contrary, *ε*_*f*_ increased with ln *** for elliptical cells. These changes in strength and ductility are affected by the strain rate hardening of components as well as the thermal effects at higher strain rates.

Aluminum and copper layers as well as the separator are shown to exhibit strain rate hardening behavior.^15,17,18^ Initial studies of our group using tensile tests indicated that polypropylene (PP) separators exhibit reduced ductility at higher strain rates.^15^ On the other hand, aluminum alloys exhibit delayed failure and enhanced ductility with increasing strain rates.^18^

Heat generated during plastic deformation can influence the ductility and strength of the cells as well. At higher strain rates, it can be assumed that an adiabatic condition exists, and the deformational heat is retained in the cell. This results in a temperature rise inside the deforming section of the cell. According to Zhang, 2017, a significant drop in separator elastic modulus is seen when increasing the temperature from 25 °C to 45 °C; however, no significant change was observed in ductility.^15^ The reported effects of the temperature on tensile properties of aluminum varies based on the grade of the material. AA5754 and AA5482 exhibited thermal softening and decreased ductility at elevated temperatures (above 150 °C),^18^ but AA5083 showed a decrease in thermal softening and an increase in ductility and fracture strain at higher temperatures.^16^ This shows the final trend in a homogenized battery cell can be affected by competing effects that higher strain rates and heat generation have on ductility of various components in each cell.

In pouch cells, the resultant effect is a reduced ductility at higher rates causing lower peak forces and smaller failure strains. For the elliptical cells on the other side, increase in peak force and failure strain shows enhanced ductility as strain rate increases. Understanding the exact underlying mechanisms that lead to this different behavior in pouch *versus* elliptical cells would require a detailed investigation of the mechanical characteristics of each component at various speeds which is the subject of future studies. The main purpose of this article was to provide knowledge on dynamic modeling of batteries, above the wide range of studies that were focused on quasi-static properties of cells and components.^19–29^

## Appendix

## Conflicts of interest

There are no conflicts of interest of any kind in the work presented in the manuscript.

## Supplementary Material

RA-009-C8RA08898E-s001
