# Supplementary material for: Dynamic impact response of lithium-ion batteries, constitutive properties and failure model
Source: RSC Adv. 2019 Jan 15;9(5):2464–73. doi: 10.1039/c8ra08898e (PMC9059972; doi:10.1039/c8ra08898e)
Supplement: RA-009-C8RA08898E-s001 [file RA-009-C8RA08898E-s001.pdf]

Electronic Supplementary Material (ESI) for RSC Advances.
This journal is © The Royal Society of Chemistry 2018

Supporting Information for 'Dynamic Impact Response of Lithium-Ion Batteries, Constitutive Properties and Failure Model'

Golriz Kermani [a,c]

Elham Sahraei [a,b,c]

[a] Electric Vehicle Safety Lab (EVSL), Department of Mechanical Engineering, Temple University, Philadelphia, PA 19122, United States

[b] Department of Mechanical Engineering, Massachusetts Institute of Technology, Cambridge, MA 02139, United States

[c] George Mason University, Fairfax, VA 22030, United States

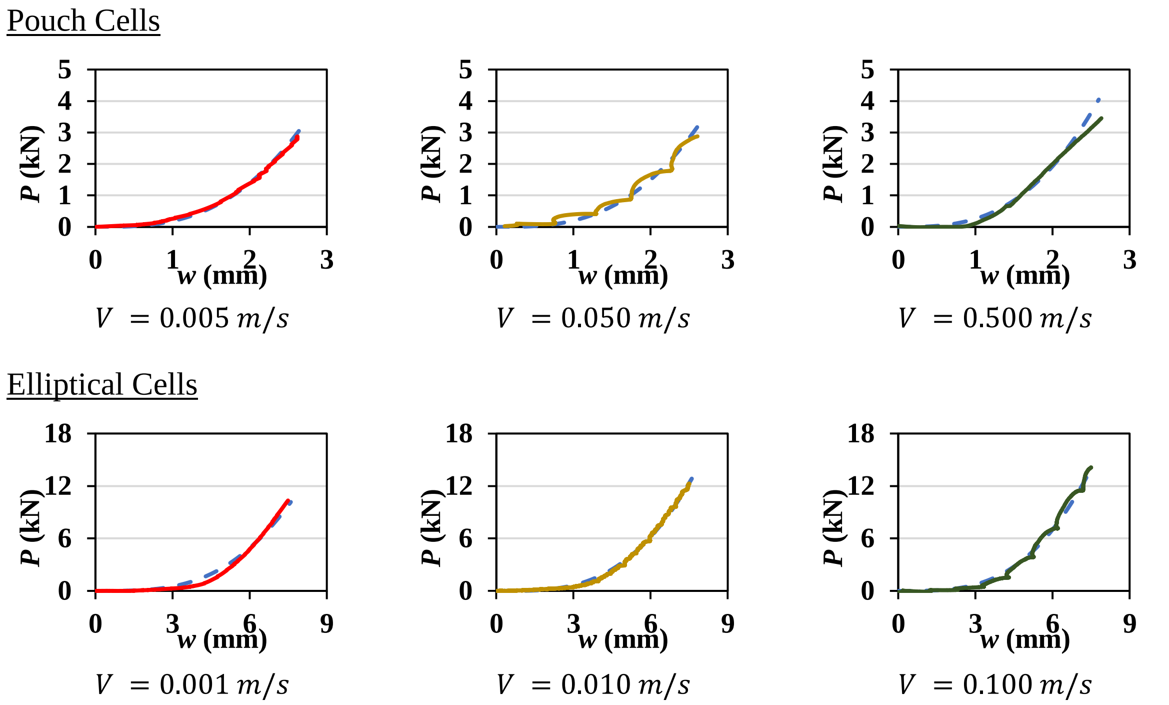

Figure S1- Analytical (dashed lines) vs. experimental (solid lines) load-displacement response of pouch cells (Top) and elliptical cells (Bottom) at different crosshead velocities

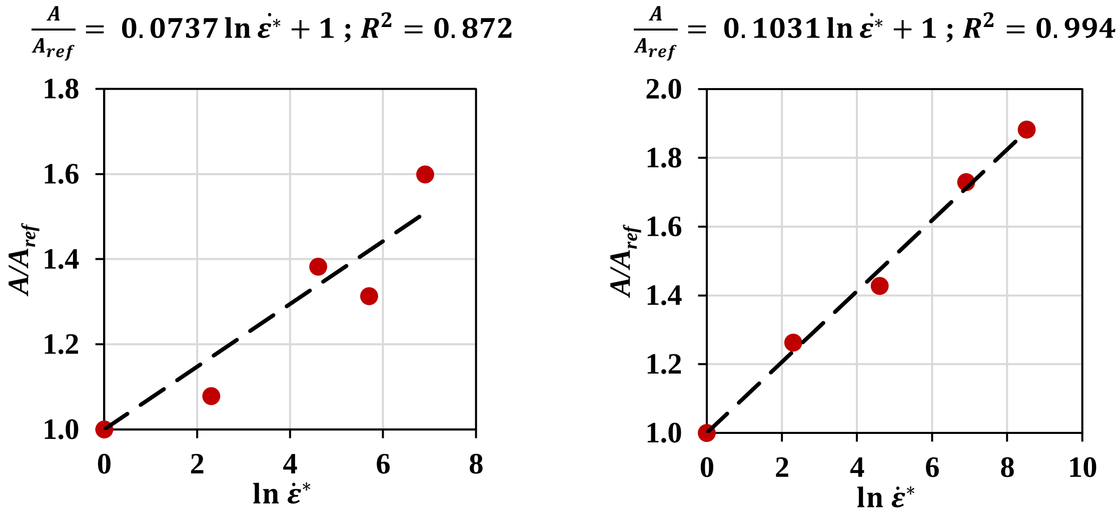

(a) Pouch Cells (b) Elliptical cells

Figure S2. Linear relationship between the normalized fit coefficients (A/A_{ref}) and $\ln \dot{\varepsilon}^*$ in a) pouch and b) elliptical cells.

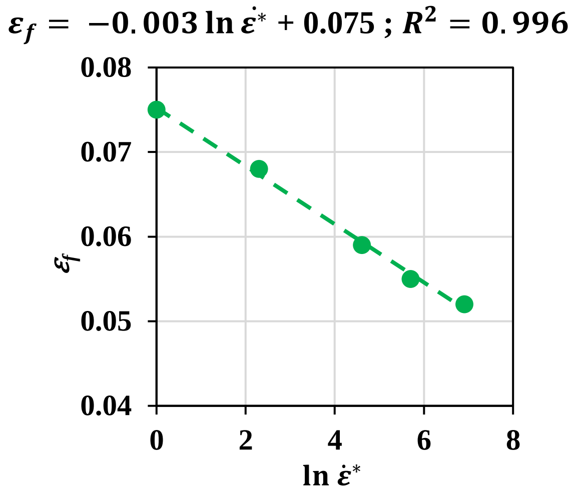

a) Pouch Cell, Mesh 1.2 mm

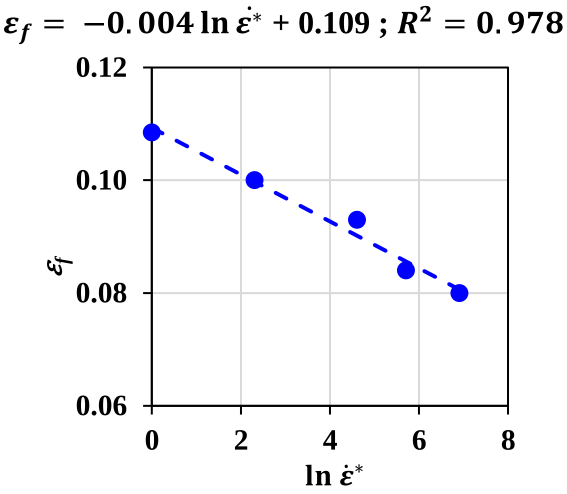

b) Pouch Cell, Mesh 2.0 mm

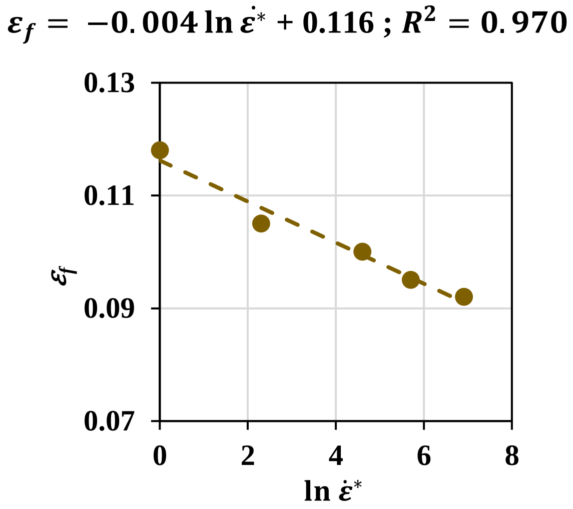

c) Pouch Cell, Mesh 4.0 mm

Figure S3. A negative linear relationship was found between ε_f and $\ln \dot{\varepsilon}^*$ in pouch cells for all mesh sizes studied (a, b, and c).
